# Periscope of Dermatology

**Published:** 1897-12-11

**Authors:** 


### Periscope of Dermatology.

(*Continued from page* 156.)

**Seborrhœa and Baldness.**—Sabouraud[1] has recently published his researches into the relations between seborrhœa, alopecia areata, and baldness. This paper is fully reviewed by Wickham[2] and others.[3] His researches about tinea tonsurans showed that the difficulty in treatment lay in the inaccessibility of the root sheath, which prevents the cure of the disease without epilation of all the infected hairs. He therefore was led to investigate the origin of alopecia, since, if this disease was due to a micro-organism, which could be isolated, it might be possible to produce a temporary baldness by injecting the toxins of the special micro-organism of alopecia. In favour of the microbial origin of alopecia it was noted that the disease always started from a central point, and that the baldness spread from this point by creeping in every direction along its circumference in the manner of a spot of oil on a fabric. He showed, furthermore, that the most pathologically active zone of the patch is situated at its confines, and that it is in this circumferential zone that the infected and broken hairs are found in the form of clubs. The micro-organism resides in the active peripheral zone, and more exactly in the dilated orifices of the hair-follicles. M. Sabouraud examined sections from a case of alopecia and seborrhœa, and was thus able to see clearly that a constant small bacillus was to be found infecting the upper part of the hair-sac. He was further able to discover that the orifices of the follicles were filled with a fatty substance which could be squeezed out on pressure, and which he called the "seborrhœic cocoon." In this seborrhœic cocoon could be found, surrounded by a crowd of other microbes, the same organism which had been demonstrated in the sections. It remained to isolate this bacillus, to cultivate it, and to demonstrate that it was the cause of alopecia areata.

The culture-medium employed by M. Sabouraud is as follows:—

| | |
|---|---|
| Peptone ... ... ... ... | 20 gme. |
| Glycerin ... ... ... ... | 20 gme. |
| Acetic acid ... ... ... ... | 5 drops |
| Water ... ... ... ... ... | 1,000 gme. |
| Gelose ... ... ... ... ... | 13 gme. |

With this medium one can obtain in many of the tubes, in the midst of the other colonies, one or two pure cultures, which are visible on the third or fourth day, the temperature being 35 deg. C. They show as pointed mounds, the colour of which is a very characteristic brick-red, on media containing glycerine, whose colour is dirty white.

A study of seborrhœa in the hairy scalp revealed (1) that the bacillus of the brick-red cultures from alopecia areata is also present in the seborrhœic plugs of the mouth of the hair and sebaceous follicles in seborrhœa, that it is there present in considerable quantity, and that it certainly is the cause of seborrhœa; (2) that consequently seborrhœa and alopecia areata have a common origin from the same micro-organism. Finally, having studied the obvious relations existing between seborrhœa and the habitual falling out of hair, Sabouraud came to the conclusion that the disseminated loss of hair in seborrhœa was the prelude of baldness.

The fact that seborrhœa and alopecia are identical is of great importance from a therapeutic point of view, as it naturally leads to the conclusion that in the initial stage of alopecia antiparasitic remedies should be employed so long as any micro-organisms can be detected on examination. The remedies used for seborrhœa, such as ichthyol, resorcin, sulphur, or oil of cade, should be thoroughly applied; later on, when no microbes can be found, irritants on the bald spot are of value. Sabouraud's conclusions are that (1) in a region affected with seborrhœa the hair dies; (2) ordinary baldness is always caused by this mechanism; (3) every bald spot is first the seat of oily seborrhœa localised to its surface; and (4) this affection is a specific microbic infection.

Brocq and Hallopean[4] do not accept the views of Sabouraud as to the identity of the cause of alopecia and of seborrhœa, and they do not admit that the latter affection is a specific microbic infection.

Crocker,[5] who has always supported the view of the microbic origin of seborrhœa, agrees thoroughly with Sabouraud's conclusions. He, therefore, urges the importance of employing antiseptics in treating seborrhœa of the scalp, and finds loretin of special value. He thinks that the seborrhœa microbe, like the trichophyton, dislikes acid media, so he includes acetic acid in the various washes. The following is recommended as a good wash: ℞.: Acetic acid, ½ to 1 oz.; resorcin, 1 drachm; eau de cologne, 2 oz.; rose water, 8 oz.; m. Glycerine, 1 drachm, can be added if the hair is dry, but it is often objected to by ladies who wish to have fluffy hair. One daily application is sufficient, but this must be repeated uninterruptedly for two months.

Skinner[6] recommends the two following hair tonics in seborrhœa capitis; the first contains quinine, the second is excellent from the small quantity of oil it contains:—

| | | |
|---|---|---|
| (1) | Quinin sulphate ... ... ... ... | 20 grn. |
| | Dilute sulphuric acid ... ... ... | qs. |
| | Tincture of cantharides ... ... ... | 1 oz. |
| | Hazelin ... ... ... ... ... | 2 oz. |
| | Glycerin ... ... ... ... ... | 1 oz. |
| | Orange-flower water ... ... ad | 8 oz. |
| (2) | Tincture cantharides ... ... ... | 14 dr. |
| | Tincture cinchona ... ... ... ... | 2 oz. |
| | Benzoin ... ... ... ... ... | 6 dr. |
| | Spirits of lavender ... ... ... | 1½ oz. |
| | Castor oil ... ... ... ... ... | 2 dr. |
| | Alcohol ... ... ... ... ... | 10 oz. |

While lanolin is the most natural grease for the hair, it is difficult to apply. If, however, it is combined with saponin, it forms a liquid lanolin, which is very well adapted for a "hair-oil." The formula given is as follows:—

| | |
|---|---|
| Lanolin ... ... ... ... ... | ½ oz. |
| Saponin ... ... ... ... ... | 4 grn. |
| Water ... ... ... ... ... | ½ oz. |
| Alcohol ... ... ... ... ... | 1 dr. |

Dissolve the saponin in the water and stir in the lanolin, previously melted. Cool, and add the spirits. This can be added to tonic preparations, or various drugs can be combined with it.

Thin[7] describes the results of histological examination of a case of alopecia areata, in which he had isolated a bacterium eight years ago; he, therefore, corroborates the views of Sabouraud, and entertains no doubt of the successful treatment of alopecia by antiparasitic remedies. The hairs should all be cut from the margin of the patch for at least half an inch, and sulphur ointment firmly rubbed into the hair follicles of this margin.

**Pemphigus.**—Miller[8] reports a case of chronic pemphigus which for twenty months affected only the mucous membrane of the oral cavity and of the epiglottis. The patient was a woman, aged 78 years, who had previously enjoyed good health. No blebs on other parts of the body, and the disease was unaffected by local treatment. The patient suffered principally from soreness of the mouth and inability to take solid food. On an attempt at mastication a fresh crop of blebs appeared, and the soreness was increased. No fœtor or salivation was present. Treatment with iron, arsenic, and strychnine was producing an improvement.

Reid[9] records the case of a man, aged 20 years, who developed an eruption of pemphigus and of boils at the same time. He was an iron rivetter, and, while hammering red-hot rivets, received a number of superficial burns on the arms. Some inflammation occurred about the burns, and when this had subsided crops of bullæ appeared on the arms.

Bacteriological examination disclosed the presence of a micrococcus, which occurred as a diplococcus with active movements; in cultures, it appeared as a coccus, diplococcus, chains, and groups, and so could not be distinguished from the staphilococcus aureus. He, therefore, thinks that the disease was due to the infection of the sores left by the burns with the micrococcus. He also refers to five other cases of pemphigus which occurred in the same farm at which this patient was working, and apparently were transmitted by contact. Later on other cases began to occur in the district in which no communication had taken place between the infected persons. A widespread epidemic occurred, and every abrasion was liable to assume an unhealthy appearance. With regard to treatment, the parts were enveloped in compresses saturated with weak formalin solution.

Beatty[10] reports three cases of epidermolysis bullosa, a disease in which bullæ form on certain parts of the body from slight injuries, and groups of subepidermic cysts are present in children. The bullæ are often hæmorrhagic, the nails are often deformed, there is no disturbance of the general health, the affection is congenital, and it is quite unaffected by drugs. The disease has usually been described as congenital pemphigus, and the author has collected a number of such reported cases, which he considers are instances of epidermolysis bullosa. His own cases occurred in a man and his two sons.

Colcott Fox[11] records the case of a woman, aged 54 years, who had suffered from pemphigus for nine years; at first indistinguishable from ordinary pemphigus, afterwards with all the characteristics of pemphigus congenitalis (epidermolysis). Bullæ arose on the least provocation, and the patient was never quite free from phlyctenæ where the corset pressed, and where the teeth touched the tongue and lips. The disease was quite unaffected by arsenic, and so the author is inclined to include the case in the group described as epidermolysis.

**Eruptions Produced by Drugs.**—Hall,[12] in a clinical lecture on this subject, divides rashes caused by drugs into two classes—(*a*) Those produced by the presence of the drug, or of some compound of it in the circulation, and (*b*) those produced by the local irritation produced by the application of the drug to the skin. With regard to the first class, the general phenomena which characterise every drug rash are: (1) The unusual parts affected; (2) polymorphism, or variety of the lesions at the same time; (3) frequent angio-neurotic characters; (4) presence of other symptoms of poisoning; (5) abatement on withdrawing the drug; and (6) possible reappearance directly it is renewed. He refers to the eruptions produced by bromides, iodides, arsenic, quinine, salicylates, and boracic acid, and states that the last-named drug produces, when applied to an abraded surface (*e g.*, in extensive burns), a bright red scarlet rash all over the body and limbs. It is difficult to diagnose from scarlatina, but there is no sore throat, vomiting, or rigor, and the temperature does not rise suddenly. The rash disappears when the boracic applications are discontinued. Walsh[13] records two cases of a dermatitis caused by gathering the plant angelica. A youth, aged 18 years, had picked angelica or cow's parsley, with his sleeves rolled up, so that the leaves and juices of the plant came freely in contact with the front of the forearms. Six days later an acute dermatitis occurred on the forearms, and blebs occurred on one side. A friend who assisted him in gathering the plant had a similar eruption. Hill[14] reports the case of a girl, aged 23, who developed an acute urticarial rash over the front of the thighs, shins, arms, cheeks, and sides of neck 18 hours after the administration of a simple soap and water enema. He considers that the rash was due to absorption of some fæcal products set free in the bowel by the liquefying action of the warm water. Balzer and Griffin[15] report two cases in which the administration of sodium cacodylate was followed by generalised desquamative erythema. Both patients had been suffering from psoriasis, and developed the disease soon after the treatment had been commenced. In one case the patient had been taking fifty grammes of cacodylic acid in the day, while in the other only fifty centigrammes was given as a dose. Taylor[16] records three cases of an eruption after mercurial inunction. In the first case this took the form of an almost universal separation of the horny layer of the epidermis after rubbing in half an ounce of blue ointment. In the second case, a man, aged 23, treated for syphilis with the ointment, developed red irritable scalp patches on the trunk, which spread over the body till the whole surface became red, dry, and scaly; this was accompanied by diarrhœa and vomiting. Tar baths and vaseline inunction gave a satisfactory result. In the third case, a girl, aged 21, after being rubbed with oleate of mercury, developed a bright scarlatiniform eruption all over her body, unaccompanied by sore throat. A few days later she began to desquamate, and then passed into a condition of acute exfoliative dermatitis, from which she died.

[1] Etude Clinique et Experimentale sur l'Origine de la Pilady Ann. de Dermatol, 1896. [2] Brit. Med. Journ., April 24, 1897, p. 1,028. [3] Amer. Med. and Surg. Bull., June 10, 1897, p. 524; Practitioner, May, 1897, p. 529; Brit. Journ. of Derm., June, 1897, p. 219. [4] Med. Week, June 25, 1897, p. 297. [5] Clin. Journ., June 2, 1897, p. 81. [6] Amer. Med. and Surg. Journ.. July 25, 1897, p. 659; Amer. Jour. of Med. Sci., May, 1897, p. 64. [7] Brit. Med. Journ., July 17, 1897, p. 127. [8] New York Med. Journ., July 3, 1897, p. 1. [9] Int. Med. Journ. of Austral., July 20, 1897, p. 441. [10] Brit. Journ. of Derm., Aug., 1897, p. 304. [11] Ditto, Sept., 1897, p. 341. [12] Clin. Journ., Sept. 1, 1897, p. 294. [13] Lancet, July 3, 1897. [14] Brit. Med. Journ., June 12, 1897, p. 1,477. [15] Med. Week, July 16, 1897

